# Clinico-demographical Profile of Pelvis and Acetabular Fracture Presenting in Tertiary Care Center of Nepal: An Observational Study

**DOI:** 10.31729/jnma.8882

**Published:** 2025-02-28

**Authors:** Ranjib Kumar Jha, Santosh Thapa, Asish Rajthala

**Affiliations:** 1 Department of Orthopedics, Nobel Medical College, Biratnagar, Morang, Nepal

**Keywords:** *acetabulum*, *epidemiology*, *fracture*, *pelvis*

## Abstract

**Introduction:**

Pelvis and acetabular fractures are complex, high-energy trauma and are often associated with life threatening injuries. The majority of them require surgical intervention and extensive rehabilitation. The epidemic figures are necessary to make plan for their management and its complications. We aim to analyze epidemiological profile of patients presented with pelvis and acetabular fracture in our center.

**Methods:**

This was observational cross-section study, analyzed on patients admitted with pelvis and acetabular fracture at a tertiary care center through emergency and outpatient department between January to December 2023. The collected parameters were age, gender, mechanism of injury, site of injury, classification of fracture, associated other injuries, management operative or non-operative, early post-operative complications, duration of hospital stay, mortality and Intensive Care Unit admission and they were analyzed. Ethical approval was obtained from Institutinal Review Committee (Reference number: 19/2024)

**Results:**

There were 48 (7.27%) pelvis and/or acetabular fracture amongst 660 patient with traumatic fractures. Pelvic fracture was 29 (4.39%) and acetabular fracture was 16 (2.42%). The median age was 38 years (IQR: 25.25-46.75) and 36 (75%) were male. Thirty three (69%) patients required surgery, 17 (35%) patients had additional surgery for associated injury and 14 (29%) required intensive care unit admission. The median hospital stay was 12.5 days and mortality rate were 2%.

**Conclusions:**

The proportion of pelvis fracture was comparable to other studies while the proportion of actebular was comparatively higher in this study.

## INTRODUCTION

Pelvis and acetabular fractures are one of the major orthopedic injuries caused by high energy trauma in road traffic accidents (RTA), crush injuries or falls from height.^1,2^ Pelvic fracture (PF) accounts 3% to 10% of traumatic injuries and is often associated with life-threatening injuries whereas acetabular fracture (AF) accounts 0.4% but it requires good surgical skill and is more associated with morbidity than mortality.^1,3,4,5^

Pelvis and acetabular fractures often require hospitalization, surgical intervention and extensive rehabilitation. In long-term, they are associated with chronic pelvic pain and gait abnormality which can cause decrease in quality of life.^6,7^

There are many studies showing clinicodemographic characteristics on pelvis and acetabular fracture in other countries, but very few have been conducted on our population. The aim of our study is to analyze the epidemiological profile of pelvis and acetabular fracture presenting to our center.

## METHODS

This was an observational cross-section study conducted at Nobel Medical College and Teaching Hospital, Biratnagar, Nepal. A retrospective data from January to December 2023 was collected following ethical approval from Institutional Review Committee (Reference number: 19/2024).

Total population sampling method was applied and all the patients that were admitted during this one year of study period were assessed for eligibility. The inclusion criteria of this study were patients of age more than 16 years admitted with diagnosis of pelvic fracture or acetabular fracture or both, who were managed in the institute for the same. Patients with incomplete preoperative radiological images and non-availability of pelvis computer tomography (CT) scan, who died immediately before investigation and who had brain dead on arrival were excluded from the study. A semistructured proforma was prepared as study tool and it was used to retrieve the data of interest from the patient medical records of our hospital.

The surgical indications of fracture fixation for pelvis fractures were unstable pelvis fracture and for acetabulum fracture were fracture displacement of >2 mm, Matta's roof angle < 45 degree or unstable/non-concentric joint reduction, unstable posterior wall fracture, acetabulum wall marginal impaction and significant fracture displacement.

Data was collected in excel sheet, pelvis and acetabular fracture was considered as independent variables and dependent variables for this study were: age, gender, mechanism of injury, site of injury, classification of pelvis and acetabulum fracture, associated other injuries, management of injuries (operative or nonoperative), early post-operative complications, duration of hospital stay, mortality and requirement of ICU admission. The data were arranged into three groups: (1) the data of the patients with pelvic fracture (PF), (2) the data of patients with acetabulum fracture (AF), and (3) the data of patients with combined PF and AF (CF). The fracture classification was determined using the X-ray and CT scan. Pelvic fractures are severe injuries often caused by high-energy trauma, such as motor vehicle accidents or falls from height. The Young-Burgess classification system categorizes pelvic fractures based on the mechanism of injury and degree of instability. Lateral compression (LC) injuries occur due to side-impact forces and are divided into LC-I (sacral compression with pubic rami fractures), LC-II (LC-I with a posterior iliac wing fracture), and LC-III (LC-II with an associated contralateral open-book or vertical shear injury, known as the "windswept pelvis"). Anteroposterior compression (APC) injuries result from frontal impact forces and range from APC-I (mild pubic symphysis widening) to APC-II (widening with sacroiliac joint diastasis) and APC-III (complete pelvic ring disruption with anterior and posterior ligament injuries). Vertical shear (VS) injuries are caused by high vertical forces, leading to significant displacement of the hemipelvis, while combined mechanism (CM) injuries exhibit features of multiple types.

Acetabular fractures, which involve the articular surface of the hip joint, are classified using the Letournel-Judet system, which divides them into elementary (simple) and associated (complex) fractures. Elementary fractures include posterior wall fractures, the most common type, followed by posterior column, anterior wall, anterior column, and transverse fractures. Associated fractures, which are more complex, include posterior column with posterior wall fractures, transverse with posterior wall fractures, and T-shaped fractures. Additionally, anterior column fractures with posterior hemi-transverse components and both-column fractures, where the acetabular dome becomes detached from the pelvis, are significant due to their impact on hip joint stability. Understanding these classification systems is crucial for guiding treatment decisions, as both pelvic and acetabular fractures often require surgical intervention to restore stability and function.^8,9^

Categorical variables are presented in terms of proportion while appropriate measures of central tendency (mean/median) and dispersion (standard deviation/interquartile range) are used for continuous variables. The data were refined and imported to SPSS software IBM SPSS Statistics for Windows, version 25 (IBM Corp., Armonk, N.Y., USA).

## RESULTS

There were 48 patients having pelvis and acetabulum fractures. The median age of patient was 38 years (IQR: 25.25-46.75), there were 36 (75%) male, 46 (95.83%) were closed fracture, 14 (29.16%) required ICU admission, 21 (43.75%) had associated injury, 13 (27.08%) had more than one surgeries and there was one (2.08%) mortality (Table 1).

There were 34 (70.83%) pelvic and/or acetabular fracture due to road traffic injury, 12 (25%) due to fall injury, 1 (2.08%) due to crush injury and 1 (2.08%) was due to various other injuries. Amongst the associated injuries, limb injury was 14 (66.67%), abdominal injury was 3 (14.29%), head injury was 2 (9.52%), chest and spine injury was 1 (4.76%) each. Young-Burgess LC1 type fracture was seen in 7 (24.14%), (Table 2).

**Table 1:** Epidemiological Characteristics of pelvis and acetabular fractures (n=48).

Variables	n (%)
**Gender**	
Male	36 (75)
Female	12 (25)
**Fracture group**	
PF	29 (60.42)
AF	16 (33.33)
CF	3 (6.25)
**Type**	
Closed fracture	46 (95.83)
Open fracture	2 (4.17)
**SICU admission**	
Yes	14 (29.17)
No	34 (70.83)
**Associated injuries**	
Yes	21 (43.75)
No	27 (56.25)
**Pelvis and acetabular surgery**	
PF	18 (37.5)
AF	12 (25)
CF	3 (6.25)
**Pelvis and acetabular(conservative)**	
PF	11 (22.92)
AF	4 (8.33)
CF	-
**Number of surgeries**	
1	25 (52.08)
>1	13 (27.08)
Mortality	1 (2.08)
**Length of hospital stay in days**	
Median(IQR)	12.5 (9.25-19.75)

PF: Pelvis fracture; AF: acetabulum fracture; CF: combined fracture; IQR: interquartile range.

**Table 2:** Distribution of pelvic fracture according to Young-Burgess classification (n=29).

Type	n (%)
LC 1	7 (24.14)
LC 2	5 (17.24)
LC 3	3 (10.34)
APC1	2 (6.9)
APC2	6 (20.69)
APC3	3 (10.34)
VS	3 (10.34)

LC:Lateral compression type; APC:Anterir posterior compression type; VS:Vertical shear type

**Table 3:** Distribution of acetabular fracture according to Letournel-Judet classification (n=16).

TYPE	n (%)
PW	4 (25)
PC	1 (6.25)
AC	1 (6.25)
AW	-
TC	2 (12.5)
**Associated types**	
PW+PC	1 (6.25)
ACPTH	2 (12.5)
TC+PW	1 (6.25)
ABC	3 (18.75)
T-type	1 (6.25)

PW:Posterior wall; PC:Posterior column; AC:Anterior column; AW:Anterior wall; TC:Transverse; ACPTH:Anterior column and posterior hemi-transverse; ABC: Associated both column.

According to Letournel-Judet classification for acetabular fracture, posterior wall fracture was seen in 4 (25%) of fractures (Table 3).

## DISCUSSION

The total of pelvis and acetabular fracture in our study was 48. The proportion of PF was 29 (4.39%), AF was 16 (2.42%) and combined pelvis and acetabular fracture was 3 (0.45%). The proportion of pelvis fracture in our study is similar to studies done in other countries which ranges from 3-10%.^3^ The proportion of acetabular fracture in our study is higher than the other study which reported 0.4%.^1^ But recent study done in Kuwait by Alhadhoud MA et all, the prevalence of pelvis and acetabulum was 5.37%, in which pelvis fracture was 3.66% and acetabular fracture was 1.71%.^10,11^ Another study in India showed increasing trend in its prevalence. The increase in prevalence may be due to rising number of vehicles use and increase in frequency of RTA. In our study, the most common mechanism of injury was RTA (70.83%) followed by fall from height (25%). In a study done in India by S. Ghosh et al, RTA was main cause of pelvis fracture in 77.3% of cases with pelvic injuries.^12^ The pelvis is strong structure which requires high energy trauma such as from RTA to develop fracture.^3,4^ In another study of epidemiological of acetabulum fracture in India showed motor vehicle accidents as main mode of injury (77.4%) followed by falls from height (19%).^11^ In our study, 95.83% of pelvis and acetabulum fractures were due to high-energy mechanism. The comparative study of operated cases of acetabulum fracture between China and USA showed 74% of fracture were due to high-energy mechanism and 26% were due to low-energy mechanism in China. Similarly, in the USA, 64% were due to high-energy and 36% were due to low-energy mechanism.^5^

Relatively younger population under 50 years and male gender are commonly involved in pelvis and acetabular fracture.^4,10,13^ In a study of acetabulum fracture by Mauffrey C et al, the mean patient age in China was 40±13 years, while the mean age in the USA centre was 44±16 years.^5^ In addition, bimodal distribution was seen in the USA centre. In both countries, the male to female ratio was 3:1. In present study, the median age of patients was 38 years and 36 (75%) of patients were male. Hence, young and economic productive adult male are prone to these fractures. Male population are major workforce and have to travel more for livelihood. So, they are more prone to have accidents. In a study done in Nepal by Paudel M et al, in RTA male gender of 20-40 years were most affected.^14^

The pelvic and acetabular fractures are often associated with injuries in other body parts because it is caused by high energy mechanism. In our study, 43.75% of patients had associated injuries and most common was fracture of upper and lower limb. The four percent of our patients had open pelvis injuries. This is comparable with epidemiological analysis in India which also demonstrated limb fractures as most common associated injuries with pelvic and acetabular fractures.^11,12^ Associated injuries to head, chest and abdomen are widely reported which is also seen in our study.^2,4,13,15^ This underlies the importance of multidisciplinary approach to these patients.

The Young-Burgess classification for pelvic ring fracture is most commonly used classification system. This is based on mechanism of injury and guides for making decision for subsequent management.^8^ In our study lateral compression force was responsible for most of pelvic ring injury and unstable fracture were more common which is similar to the findings in other literature.^16,17^ In this study, we found isolated posterior wall (25%) as most frequent fractures followed by associated both column fracture (18.75%) which was similar to other study.^1,11^ In another study, posterior wall fracture was seen in 30% in China and 32% in the USA followed by associated both column fractures (21% and 17% respectively).^5^ We did not find any case of anterior wall fracture. In one of the largest epidemiological series of surgically treated acetabular fractures in India, the anterior wall fracture was least common (0.95%).^11^ The mortality rate in our study was 2.08% in pelvis fracture group, but there was no any mortality in acetabular fracture group. The lower mortality in our study may be because of exclusion of patients from study who were brought dead and those who died in emergency before any investigation. This figure is lower than the other studies which showed wide range of mortality rate of 3-60%.^3,4,18^ Our findings are similar to study done by Cuthbert R et al.,^19^ in which they found that intensive care admission was necessary in 43%, with median hospital stay of 13 days. Furthermore, 48% required pelvic and acetabular surgery, with 38% needing additional surgery for the other orthopedic injuries. Operative management was required in 43% of acetabular fracture in United Kingdom and in 22% of pelvis fracture in Germany.^1,2^ The literature from United Kingdom have demonstrated that pelvic and acetabular fractures needed more intensive care admissions (24.5% vs 11.7%) and longer inpatient stays (15 vs 8d) than any other high energy traumatic injuries.^4^

In a study in Germany by, O Hauschid et al,^20^ they found mortality rate of 8.20% and most deaths in patients with pelvic fractures are not caused by pelvic fracture itself but are due to associated injuries. In another study in UK, the 3-month cumulative mortality of patients with pelvic ring injuries was 14.2% versus 5.6% in non-pelvic injuries. They also found that age, early physiologic derangement and presence of other injuries (head or trunk) were associated with increased risk of mortality.^4^

In our study, intensive care admission was required in 29.16% of patients and the median (IQR) length of hospital stay was 12.5(9.25-19.75) days. The 68.75% of patients required pelvic or acetabular surgery, more frequent with acetabular fracture, 40% required additional surgery and 27.08% needed more than one surgery. It shows that pelvic and acetabular fractures need considerable hospital resources and causes significant socioeconomic burden to the patients.

The present study gives an idea about the demographic factors of pelvic and acetabular fractures, their incidence and associated injuries and their impact. However, there are some limitations. Ideally epidemiological study would be complemented by outcomes data. As this is retrospective study, various outcomes could not be measured. This is single center study, so sample size is less. We recommend to start a multi-center computerized trauma registry in our country. Further prospective studies are required to specify the surgical management planning and measure outcomes after surgery and its complications.

## CONCLUSIONS

The proportion of PF was comparable to other studies while the proportion of AF was comparatively higher in this study. The most common pelvis fracture is LC type whereas, posterior wall fracture is most common acetabular fracture. The majority of these fracture required surgical treatment.
